# Left, Then Right Internal Carotid Artery Dissection: A Case Report

**DOI:** 10.5811/cpcem.21189

**Published:** 2024-10-22

**Authors:** Jeffrey M. Kalczynski, John Douds, Michael E. Silverman

**Affiliations:** * Morristown Medical College, Department of Emergency Medicine, Morristown, New Jersey; † Sidney Kimmel Medical College, Philadelphia, Pennsylvania

**Keywords:** stroke, carotid dissection, artificial intelligence, case report

## Abstract

**Introduction:**

We present a unique case of a patient who presented to the emergency department with stroke-like symptoms found to have a spontaneous, left-sided internal carotid artery dissection (ICAD).

**Case Report:**

The patient was treated successfully with thrombectomy and subsequently developed contralateral symptoms caused by a right-sided ICAD. This was managed with a second contra-lateral thrombectomy. The patient’s course was complicated by persistent and mild hypotension, postulated to be secondary to bilateral carotid baroreceptor trauma from the dissections.

**Conclusion:**

This case highlights the importance of close neurological monitoring for patients, preferably in a neurologic critical care setting, during and after invasive treatments such as systemic thrombolytic administration or mechanical thrombectomy. In this case, identifying the patient’s subsequent development of contralateral symptoms in a timely fashion was key to his positive outcome. An additional factor that had a positive impact on this outcome was the use of artificial intelligence software, which assists in determining whether thrombectomy may be indicated prior to receiving a formal radiologist read on computed tomography angiography/perfusion studies. Artificial intelligence technology such as this has great potential to augment and expedite patient care.

## INTRODUCTION

As a major cause of death in the United States, and a significant cause of disability, stroke management is critically important to the practice of emergency medicine. Ischemic stroke due to arterial dissection represents a subset of pathology that can cause large vessel occlusions, a devastating condition with major potential impacts to morbidity and mortality. The pathophysiology of internal carotid artery dissection (ICAD) involves a defect in the intima of the arterial wall, which can develop spontaneously as the result of trauma or due to multifactorial causes linked to cardiovascular risk factors.^1^ This defect allows blood products to enter the space between the intima and the media, expanding the intima into the vessel’s lumen, which reduces blood flow and can lead to thromboemboli.^2^ Internal carotid artery dissection is a major cause of morbidity and mortality in young to middle-aged patients, accounting for approximately 25% of ischemic strokes in these populations.^1^ The incidence rates for spontaneous ICAD “have been previously reported to be 2.6–2.9 per 100,000.”^3^

We report the case of a patient who suffered an atraumatic, spontaneous ICAD, which was treated with thrombectomy, and who then subsequently developed contralateral symptoms. Upon reevaluation in the neurological intensive care unit (ICU), the patient was found to have a secondary contralateral, atraumatic, spontaneous ICAD. There are no reported cases like this in the literature to our knowledge. This case emphasizes the importance of close monitoring and reassessment of critically ill patients in the neurologic critical care setting, as well as the aggressive nature of diagnosis and treatment that this subset of patients requires.^1^,^4^ Interestingly, our patient developed persistent mild hypotension after his bilateral dissections, presumably secondary to baroreceptor trauma from the dissection. There is a report of dysautonomia after bilateral dissections,^5^ and others with stroke-like symptoms and dysarthria,^3^,^6^–^8^ but none with isolated persistent hypotension.

## CASE REPORT

This is a case of a 60-year-old male with a history of hyperlipidemia presenting to the emergency department complaining of right-sided paresthesias and clumsiness as well as progressive word-finding difficulties with an onset time of 37 minutes prior to evaluation. The patient’s wife provided the history as he was only able to say the word “yes” when questioned. Blood glucose was within normal limits, and an initial National Institutes of Health Stroke Scale was as follows: 8 Total (2: Loss of Consciousness questions, 2: Loss of Consciousness Commands, 1: Sensory, 2: Best Language, 1: Extinction and Inattention.) Code stroke was immediately activated, and a non-contrast computed tomography (CT) of the head was without signs of hemorrhage. A CT angiography of the head and neck with perfusion was concurrently performed and revealed a large, left-sided ischemic penumbra.

**CPC-EM Capsule**

What do we already know about this clinical entity?

*Internal carotid artery dissection (ICAD) can cause cerebral ischemia and neurological symptoms. It is frequently treated with mechanical thrombectomy.*

What makes this presentation of disease reportable?

*This patient suffered ICAD twice in one day on opposite sides of the bod*y.

What is the major learning point?

*Patients in the emergency department require close monitoring and reassessment, especially during and after interventions such as thrombolytic therapy and endovascular treatments.*

How might this improve emergency medicine practice?

*Artificial intelligence programs can be integrated with standard emergency care to improve patient outcomes.*

There were no exclusions to thrombolytic therapy, and tenecteplase was administered. There was a significant improvement in neurological function five minutes after tenecteplase, but a repeat exam shortly after revealed return of symptoms. Artificial intelligence (AI) software (Rapid AI, San Mateo, CA) indicated that a mechanical thrombectomy could have been indicated; neurosurgery was consulted and agreed that thrombectomy was indicated. The patient was taken to the neurosurgery suite, where a left-sided mechanical thrombectomy was performed. He was found to have suffered a left-sided ICAD.[Table t1-cpcem-8-365]

Shortly after arriving in the neurological ICU status post-mechanical thrombectomy, the patient was noted to have developed new left-sided neurological symptoms, prompting repeat CT angiography with perfusion, which revealed a new right-sided perfusion deficit (Image 2). The patient was urgently returned to the thrombectomy suite for contralateral thrombectomy and was found to have suffered a spontaneous, right-sided ICAD.

On return to the neurological ICU, he was again with improved neurological function, but with persistent mild hypotension requiring vasopressor initiation. He was eventually transitioned to midodrine and was subsequently discharged to subacute rehab. It is theorized that the hypotension was secondary to bilateral carotid baroreceptor damage after the bilateral dissections.

## DISCUSSION

The sequential, bilateral ICADs presented in this case represent a rare presentation. Unilateral headache and stroke-like symptoms have been reported before—once in a 43-year-old female found to have bilateral ICADs as a result of trauma^3^ and once in a 38-year-old female with spontaneous bilateral ICADs.^6^ Other presentations of ICAD including dysphagia, hoarseness, and dysarthria have been reported in middle-aged males.^7^,^8^

Uniquely, our patient developed persistent mild hypotension status post-bilateral mechanical thrombectomy of the carotid arteries, requiring vasopressor support. While dysautonomia involving episodic bradycardia with hypotension has been previously reported in a 49-year-old female with bilateral ICADs,^5^ there have been no previously reported cases of persistent dysautonomia as in this case we report. The prevailing theory is that damage to the carotid baroreceptors occurred bilaterally, either secondary to the dissections or iatrogenically by the mechanical thrombectomies, with resultant thromboemboli.

The carotid baroreceptors located in the carotid sinuses are responsible for sensing blood pressure via stretch receptors and afferently transmitting these signals along the glossopharyngeal nerve to the midbrain; then the midbrain sympathetically alters heart rate and contractility to adjust pressure accordingly, a process known as the carotid sinus baroreflex.^9^ Baroreceptors are also present in the aortic arch, which monitor pressure and transmit signals via the vagus nerve to the medulla, working in conjunction with the carotid sinuses to achieve homeostasis; these baroreceptors are thought to be more sensitive to increased pressure, while the carotid sinuses are sensitive to both increased and decreased pressure.^10^ Therefore, if the carotid sinuses were rendered inoperative, as proposed in this patient, the aortic arch baroreceptors may have been unable to initially sense the change and induce a compensatory response, resulting in persistent mild hypotension. In this case, the patient’s hypotension initially required pressor support with low-dose norepinephrine bitartrate, which was eventually titrated down, and the patient was switched to oral midodrine for further outpatient management.

**Image 1:**
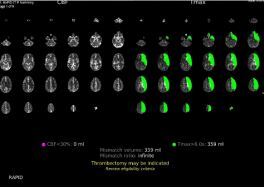
Initial computed tomography perfusion study depicted on the left with overlaid deficits in green as identified by artificial intelligence software on the right.

The CT angiogram with perfusion studies obtained for this patient’s diagnostic evaluation were critical in the timely recognition and treatment of the pathology. The perfusion studies were particularly useful in identifying the brain tissue receiving reduced blood flow as a result of each individual ICAD. The initial perfusion study, as shown in Image 1, depicts 30 slices of the patient’s brain from the CT angiography on the left side, while the right side depicts the same slices with a green overlay depicting areas with reduced perfusion as identified by AI.^11^

**Image 2:**
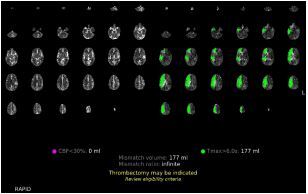
Repeat perfusion study for evaluation of contralateral symptoms on the left with overlaid deficits in green as identified by artificial intelligence software on the right.

The repeat CT angiography with perfusion study in Image 2 reflects the perfusion defects on the contralateral side at the time of the patient’s second spontaneous ICAD.

These AI-generated overlays assisted the emergency, neurological, and neurosurgical teams in early identification of a perfusion deficit of the regions experiencing reduced perfusion, and of the location of the perfusion deficit’s source.

The integration of AI software into the evaluation of radiological imaging studies is becoming increasingly common.^12^ The development of AI with the ability to analyze CT imaging has been studied^13^ and has been deemed non-inferior in its evaluation of brain ischemia.^14^ Furthermore, AI software integrated into imaging systems has been shown to improve the ability of non-experts to evaluate imaging.^15^ The RapidAI software we used with this patient analyzed the CT angiogram with perfusion study and determined whether thrombectomy was indicated. In Images 1 and 2, the decision made by the AI software can be seen in yellow lettering at the bottom of the studies, indicating that the software correctly identified the need for thrombectomy in both studies. This software allows the emergency physicians and neurosurgical teams to coordinate care with interventional neuroradiology in preparation for thrombectomy, even before radiology had provided an official read of the images. The integration of AI software has the potential to decrease time to thrombectomy in patients with perfusion deficits and lead to improved patient outcomes.

## CONCLUSION

This case is notable for the occurrence of bilateral, spontaneous ICADs with resulting persistent mild hypotension, emphasizing the need for close monitoring of patients in the neurological ICU. Patients with bilateral ICADs and/or bilateral thrombectomies should be monitored for signs of dysautonomia due to the potential for a deficient carotid baroreflex with insufficient aortic baroreceptor compensation. Computed tomography angiography with perfusion can be instrumental in the early identification and localization of perfusion deficits, especially when image evaluation is assisted by AI software. Further studies should evaluate the integration of such software into image evaluation to measure its ability to reduce time to treatment for patients with brain ischemia which could affect morbidity and mortality in this patient population.

## Figures and Tables

**Table t1-cpcem-8-365:** Timeline of events from initial presentation to second thrombectomy.

Time	Event
06:45 am	Symptom onset
07:12 am	Patient presentation to emergency department
07:22 am	Code stroke activation
07:45 am	Normal CT head prelim read by radiology
08:02 am	Tenecteplase administration
08:10 am	Perfusion abnormality noted by AI software
08:12 am	Neurosurgery consulted
09:15–10:30 am	Thrombectomy for left ICAD
12:30 pm	Development of contralateral symptoms
12:30 pm	Thrombectomy for right ICAD
3:00 pm	Hypotension identified
3:15 pm	Norepinephrine bitartrate support started
8:30 am (next day)	Transitioned to midodrine

*AI*, artificial intelligence; *CT*, computed tomography; *ICAD*, internal carotid artery dissection.
